# A comparison of the burden of cancers between 1990 and 2019 in Iran: A national and subnational study

**DOI:** 10.1371/journal.pone.0309699

**Published:** 2025-02-25

**Authors:** Mitra Darbandi, Zahra Khorrami, Ali Karamoozian, Omid Aboubakri, Mahsa Miryan, Leila Rezakhani, Fatemeh Khosravi Shadmani

**Affiliations:** 1 Research Center for Environmental Determinants of Health (RCEDH), Health Institute, Kermanshah University of Medical Sciences, Kermanshah, Iran; 2 Student Research Committee, Kermanshah University of Medical Sciences, Kermanshah, Iran; 3 Ophthalmic Epidemiology Research Center, Research Institute for Ophthalmology and Vision Science, Shahid Beheshti University of Medical Sciences, Tehran, Iran; 4 Modeling in Health Research Center, Institute for Futures Studies in Health, Kerman University of Medical Sciences, Kerman, Iran; 5 Department of Biostatistics and Epidemiology, Kerman University of Medical Sciences, Kerman, Iran; 6 Environmental Health Research Center, Research Institute for Health Development, Kurdistan University of Medical Sciences, Sanandaj, Iran; 7 Fertility and Infertility Research Center, Health Technology Institute, Kermanshah University of Medical Sciences, Kermanshah, Iran; Mashhad University of Medical Sciences, IRAN, ISLAMIC REPUBLIC OF

## Abstract

**Background:**

Cancer is a rapidly increasing global problem, and one of the leading causes of burden and mortality. This study aims to compare the burden of cancer in Iran between the year 1990 and 2019.

**Methods:**

We used Global Burden of Disease data on cancer from 1990 to 2019 by province, year, age group, and sex. We then estimated the trend of age standardized mortality and Disability-Adjusted Life Years (DALYs) of the cancers by sex. Age pattern and geographical variation in the ranking of cancers were assessed at national and sub-national levels from 1990 to 2019.

**Results:**

The mortality rate decreased from 102 (95% UI: 91, 111) to 96 (95% UI: 88, 103) per 100000 population. Additionally, the DALYs rates decreased from 2619 (95% UI: 2357, 2852) to 2321 (95% UI: 2116, 2497) per 100000 between 1990 and 2019. Both of the mortality and DALYs rate from cancers increased with age. These indicators were significantly higher in men than in women across all age groups. Consequently, the mortality rate and DALYs per 100,000 of cancers were higher in the northwest and northeast of Iran. Notably, stomach cancer was identified as the leading cause of cancer mortality in 23 provinces of Iran in 2019. The highest percentage change of DALYs per 100,000 rate between 1990 and 2019 was observed for malignant skin melanoma, stomach cancer, and cervical cancers with rate of −41.1, −40.1, and −38.4, respectively.

**Conclusion:**

Overall, the mortality and DALYs per 100,000 rates of all cancers for both sexes in Iran have decreased between 1990 and 2019. However, there is an increasing trend in types of cancers, such as pancreatic, ovarian, and breast cancers.

## Introduction

Cancer, as one of the main causes of mortality, is rapidly increasing worldwide [1]. In 2015, World Health Organization (WHO) reported that cancer was the leading or second-leading cause of death among people aged 70 years and older in 91 out of 172 countries. According to the reports, cancer ranked as the third or fourth leading causes of deaths in 22 countries [2]. The burden of cancers, measured by both Disability-Adjusted Life Years (DALYs) and mortality, is not uniform across the world [3]. It has changed over time and varies geographical regions in both developed and developing countries. These changes are influenced by risk factors such as aging and socioeconomic status [2,4].

In Iran, cancer is estimated to be the second most significant health issue and the third leading cause of death, following cardiovascular diseases and injuries, with mortality rates of 65 per 100,000 for men and 41.1 per 100,000 for women [5]. Recently, the lifestyle change among the Iranian population have become more pronounced due to industrialization and modernization [6].

Mortality is an important indicator for understanding cancer patterns and trends, as well as for developing interventions and assessing policies [7]. In addition to mortality, DALYs is another measurement of health loss due to illness, reflecting the ideal condition where people live without health problems until old ages [8]. DALYs are combines Years of Life Lost (YLL) and Years Lived with Disability (YLD) [9]. YLL is estimated using life expectancy and a standard life table based on the age of death. YLD is calculated using the prevalence of a disease, its major morbidity outcomes, and a weighted value of severity [10]. Therefore, analyzing point values and trend in DALYs help compare the health burden of different regions.

Recently, Iran has faced rapid ageing and is also experiencing an economic recession, leading to increasing the non-communicable diseases risk factors. Therefore, understanding the status of burden of cancer in Iran from 1990 to 2019 is crucial and can provide sufficient evidence for planners and decision-makers in health programs. This study aims to compare the burden of cancers at the national and subnational levels in Iran between 1990 and 2019.

## Methods

### Study location

Iran is a country located in the western region of Asia. It is bordered by Iraq, Turkey, Azerbaijan, Armenia, Turkmenistan, Afghanistan and Pakistan. The country covers an area of 1,648,195 km^2^, making it the second-largest country in Western Asia and the fourth-largest country on the continent. According to the latest projections of the United Nations Population Division, as of August 10, 2022, the population of Iran has been estimated to be 86,132,620 [11]. The WHO data, published in 2020, reported life expectancy in Iran to be 77 years [12]. Iran is subdivided into thirty-one provinces with different socio-economic, cultural, and geographic variations.

### Data sources and study population

The Global Burden of Disease Study (GBD), as a comprehensive investigation, evaluates 286 causes of death, 369 diseases and injuries, and 87 risk factors in 204 countries and regions [13]. The GBD includes the indicators of mortality, YLL, YLD, DALYs, and countries’ incidence and prevalence data in number, rate and percent [13]. In brief, in the GBD study, indicators of any cause and attributable mortality and burden of risk factors by location, age groups, and sex were measured from 1990 to 2019. Estimation of the 2019 GBD based burden of diseases, injuries, and risk factors has been reported elsewhere [14,15]. Mortality data were collected from vital registration systems and verbal autopsies. For locations where mortality data are unavailable, mortality was estimated. Estimated mortality data were derived by multiplying high-quality cancer registry incidence data by the corresponding, independently modelled, mortality-to-incidence ratio (MIR). Estimates of the incidence of each cancer type were obtained by dividing modelled mortality estimates by MIR. For estimation of YLDs, the total prevalence was divided into phases of cancer treatment, and then YLD estimates were calculated as the weighted sum of prevalence, where prevalence was estimated from cancer survival data modelled on the basis of MIR [15]. Disability weight ranged from 0 (full health) to 1 (death) [15]. YLLs were estimated as the weighted sum of the number of deaths by age, where the weights were the standard life expectancy at each age-group of deaths [15]. DALYs were calculated as the sum of YLDs and YLLs. In this study, cancer data were obtained from the Iranian annual mortality data based on vital registration, verbal autopsy and cancer registry, and DALYs calculated by age groups, sex, province, and cancer type from GBD 2019. These data were analyzed from 1990 to 2019. In this study, the 29 types of cancers were studied in 31 Iranian provinces

### Statistical Analysis

The overall burden of disease was assessed using the DALYs, a time-based measure that combines YLLs due to premature mortality and years of life lost due to time lived in states of less than full health, or years of healthy life lost due to disability (YLDs). One DALY represents the loss of the equivalent of one year of full health. The age-standardized mortality rate (ASMR) and DALYs rate were weighted to be averages of the age-specific mortality and DALYs rates per 100,000 persons. Sex and age-specific differences in cancer represent a health disparity. The type of cancer, diagnostic methods and treatment decisions are different according to the gender and age group of the patients. Also, the risk factors for cancer (smoking, sexual behavior, etc) are different in men and women and age groups. In this paper, the trend of cancer-related age-standardized mortality and the DALY rate for males and females have been studied from 1990 to 2019. Also, we described the age pattern of mortality rate and DALYs for all types of cancers in 1990 and 2019 by sex. On the other hand, the geographical distribution of mortality and DALYs rates were shown by maps in 1990 and 2019.

We have conducted a 1990 and 2019 comparison between the ranks and proportions of cancers responsible for the mortality rate and DALYs at national and subnational levels in Iran. Also, the rank of cancers at the subnational level was assessed. Additionally, the percent changes were calculated for mortality rates and DALYs. It is worth mentioning that all analyses were performed using R software version 4.0.2 (2020.06.22).

## Results

The main results of this study indicate that the trend of age-standardized mortality rates and disability-adjusted life years (DALYs) due to all cancers decreased from 1990 to 2019. Mortality rates were significantly higher in men than in women across all age groups, with the highest rates observed in individuals older than 70. In contrast, the greatest DALYs occurred in those over 50. Overall, mortality rates and DALYs per 100,000 exhibited geographical variation, being higher in the Northwestern and Northeastern provinces compared to other regions. Notably, stomach cancer was the leading cause of cancer mortality in 23 provinces of Iran in 2019.

**Fig 1:**
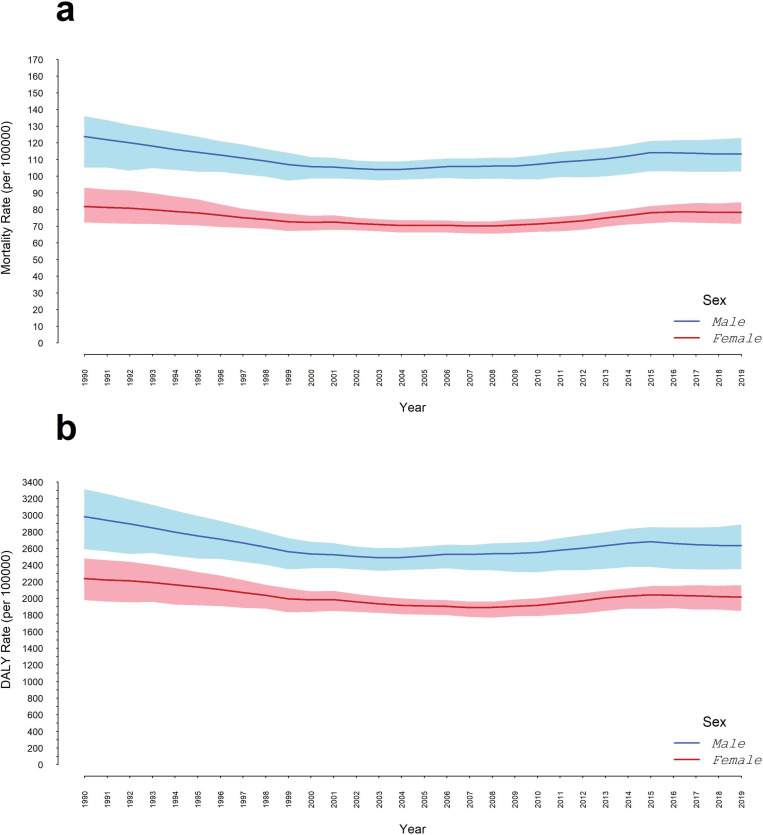
The age-standardized trends of all cancers in Iran. a) The Mortality Rate; b) DALYs per100000 rate between 1990 and 2019 in Iran.

The age-standardized trends of mortality and DALYs rates per 100,000 for all cancers in Iran are illustrated in Fig 1. From 1990 to 2019, the mortality rate for men decreased from 123.7 (95% UI: 135.9, 105.3) to 113.2 (95% UI: 122.8, 102.8), while for women it fell from 81.7 (95% UI: 93.1, 72.2) to 78.2 (95% UI: 84.4, 71.5) (Fig 1a). DALYs per 100000 also declined for men, from 2983.7 (95% UI: 3312.3, 2590.0) to 2632.5 (95% UI: 2885.8, 2353.6), and for women, from 2236.9 (95% UI: 2479.0, 1977.3) to 2013.7 (95% UI: 2159.97, 1849.4) (Fig 1b).

**Fig 2:**
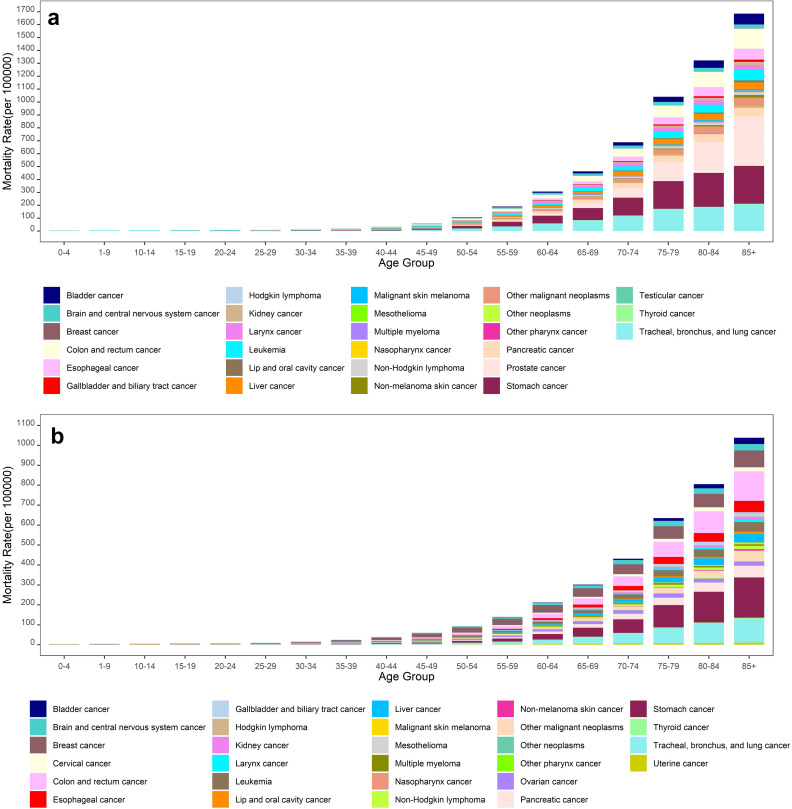
Mortality rate for different types of cancers in Iran, 2019, a) Men; b) Women.

The highest mortality rates for various cancer types were found in individuals over 70 years old, with men experiencing significantly higher rates than in women across all age groups (Fig 2). Among men aged 70 or older, prostate cancer was the leading cause death in Iran in 2019, followed by stomach and tracheal, bronchus, and lung cancers (Fig 2a). For women in the same aged group, stomach cancer was responsible for the highest mortality in Iran in 2019, followed by tracheal, bronchus, lung and colon and rectum cancers (Fig 2b).

**Fig 3:**
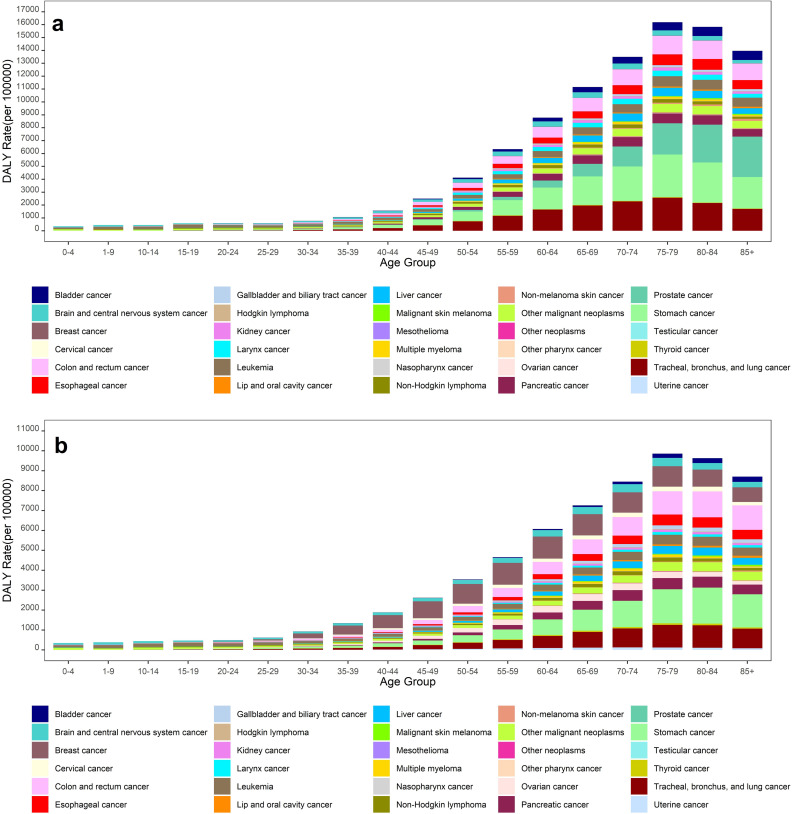
DALYs for different types of cancers in Iran, 2019, a) Men; b) Women.

The greatest DALYs rates were observed in individuals over 50, with men again significantly higher rates in all age groups (Fig 3). For men over 50, prostate, and stomach cancers w contributed the most to DALYs in Iran in 2019, followed by cancers of the colon, rectum, tracheal, bronchus, and lung (Fig 3a). Among women over 50, the highest DALYs were attributed to stomach cancer, tracheal, bronchus, lung, and colon and rectum cancers in 2019 (Fig 3b).

**Fig 4:**
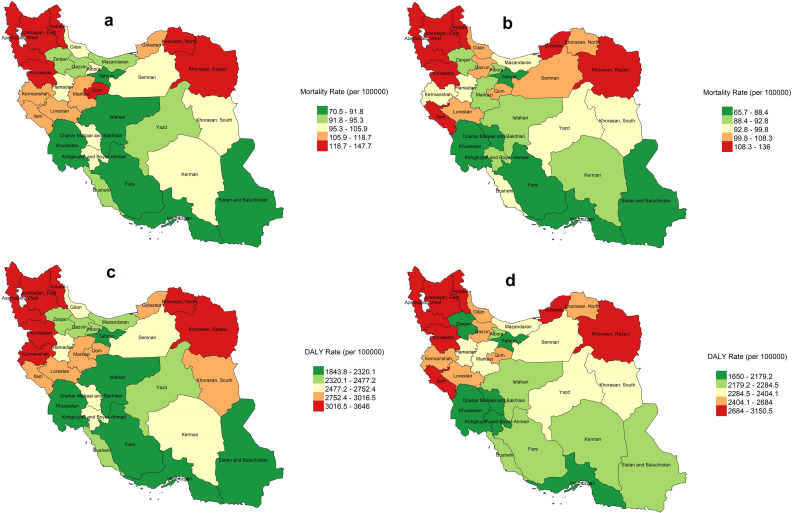
Distribution of age-standardized total cancer in the provinces of Iran, 1990 and 2019. a) Mortality rate in 1990; b) Mortality rate in 2019; c) DALY rate in 1990; d) DALY rate in 2019.

Fig 4 shows the distribution of mortality and DALYs per 100,000 rates in the provinces of Iran for all cancers in 1990 and 2019. In 1990, the provinces with the highest mortality rates included Razavi Khorasan, North Khorasan, Qom, Ardabil, East Azerbaijan, West Azerbaijan, and Kurdistan (Fig 4a). By 2019, the highest rates remained in Razavi Khorasan, Golestan, Ilam, Ardabil, East Azerbaijan, West Azerbaijan, and Kurdistan (Fig 4b). Overall, mortality and DALYs per100, 000 rates due to cancers were higher in the Northwestern and Northeastern provinces compared to others. The distribution of DALY rates closely matched that of mortality rate in Iran (Fig 4c, d).

**Fig 5:**
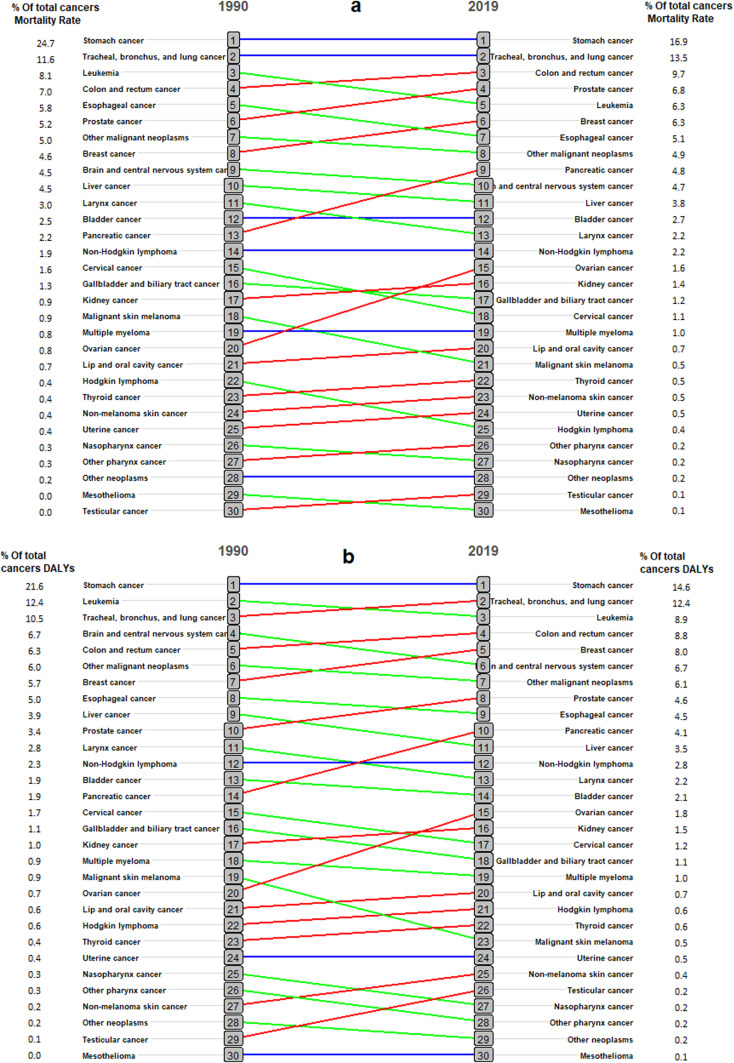
Changes in mortality and DALYs per100,000 rates by cancer types from 1990 to 2019 in Iran; a) Mortality rate; b) DALYs per100000 rate.

When analyzing cancer mortality rates from 1990 to 2019, pancreatic cancer rose from 13th to 9th place, while ovarian cancer increased from 20th to 15th place. In contrast cervical cancer, malignant melanoma of the skin, larynx, Hodgkin’s lymphoma, and leukemia showed a decline by 2019 compared to 1990 (Fig 5a). In terms of DALYs per 100,000 breast cancer moved from 7th to 5th place, followed by pancreatic cancer, which increased and has moved from 14th to 10th place, and ovarian cancer from 20th to 15th place. Malignant skin melanoma improved from 5th to 4th place. Additionally, cancers of the brain, central nervous system, liver, and cervical decreased in rank from 1990 to 2019 (Fig 5b).

**Fig 6:**
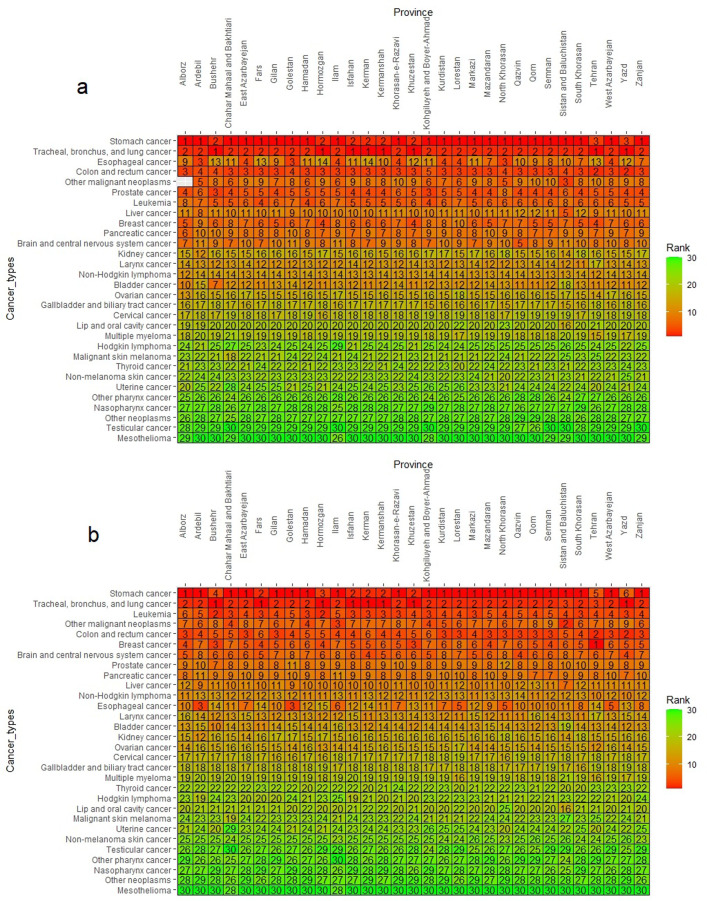
Ranking of age-standardized mortality rates and DALY in each province of Iran for cancers, 2019. a) Mortality rate; b) DALY.

In 2019, stomach cancer was the leading cause of cancer mortality in 23 provinces of Iran. Colon and rectum cancer ranked second in mortality in Yazd and Tehran provinces. While prostate cancer was the third most common cause of death in Bushehr and Kohgiluyeh and Boyer-Ahmad provinces (Fig 6a). The DALY rate due to cancers in Iran displayed a similar pattern to mortality in 2019. The DALYs per 100,000 rates for stomach cancer were highest in all provinces except Tehran, Yazd, Bushehr and Hormozgan. Prostate cancer ranked first or second in the DALY rate caused by cancer in all provinces except Tehran, Sistan, and Baluchestan province. Esophageal Cancer ranked third in Ardabil and Golestan, but had lower ranks (5th to 14th) in other provinces (Figure 6b). Table 1 presents the percentage changes in DALYs per 100,000 and the mortality rate for different types of cancers in Iran between 1990 and 2019. The largest changes in DALY were observed for malignant skin melanoma, stomach, and cervical cancers with percentage changes of −41.1, −40.1, and −38.4, respectively. The smallest changes in mortality were also seen in malignant skin melanoma, nasopharynx, stomach, and cervical cancer.

**Table 1:** Percent of change for mortality and DALYs per100000 rates by cancers in Iran (1990 to 2019).

Cancer type	Percent of change (1990 to 2019)	
	Mortality rate	DALY
Stomach cancer	−36.0	−40.1
Tracheal, bronchus, and lung cancer	8.9	4.0
Leukemia	−27.1	−36.7
Colon and rectum cancer	29.9	24.8
Esophageal cancer	−23.0	−27.8
Prostate cancer	22.0	16.9
Breast cancer	16.5	18.2
Other malignant neoplasms	−2.1	−4.7
Brain and central nervous system cancer	−1.1	−10.7
Liver cancer	−20.4	−20.9
Larynx cancer	−31.8	−35.5
Bladder cancer	1.9	0.7
Pancreatic cancer	117.0	107.5
Non-Hodgkin lymphoma	10.2	8.0
Cervical cancer	−35.5	−38.4
Gallbladder and biliary tract cancer	−13.0	−15.7
Multiple myeloma	−0.3	−0.7
Kidney cancer	39.8	28.1
Malignant skin melanoma	−42.9	−41.1
Ovarian cancer	92.0	84.4
Lip and oral cavity cancer	−1.3	−5.2
Hodgkin lymphoma	−14.2	−6.5
Uterine cancer	15.7	19.5
Thyroid cancer	15.1	26.4
Non-melanoma skin cancer	28.5	23.1
Nasopharynx cancer	−36.6	−36.4
Other pharynx cancer	−21.7	−24.4
Other neoplasms	−18.3	−13.9
Testicular cancer	176.2	176.1
Mesothelioma	31.3	28.3

## Discussion

In this study, we estimated the burden of all cancers in the Iranian population from 1990 to 2019 using the latest GBD 2019 data. This research presented a slight decrease in mortality rates and DALYs per 100,000 for both genders over the last three decades. The mortality rate per 100,000 decreased by 8.5% for men and 4.3% for women from 1990 to 2019. Additionally, DALYs per 100,000 declined by 11.7% and 9.9% during the 30 years in men and women, respectively.

This study showed that the highest mortality rate and DALY for different types of cancers were observed in the age group over 70 and age group over 50, respectively. These findings are consistent with those reported in other studies [16–18]. Our results revealed that the mortality rate and DALY in men were substantially higher than in women in the age group over 50 years. Among men, prostate cancer had the highest mortality rate and DALY in 2019, followed by the stomach, tracheal, bronchus, lung cancers, colon, and rectum.

An article analyzing data from 48 Asian countries from 1990 to 2019, using data from the Global Health Data, found that the ASIR and prevalence of prostate cancer increased in all countries. Additionally, mortality rates and DALY also increased in most of these countries.

Their data suggested that countries in Western Asia had the highest mortality and DALY [19]. The GBD 2015, indicates a decreasing trend in the prostate cancer mortality rate in high-income countries. However, DALYs attributable to prostate cancer increased by 90% from 1990 to 2015 [20]. Recently, two meta-analyses reported an ASR of 9.1 per 100,000 [21] and 8.7 per 100,000 [22] for prostate cancer incidence in Iran. Prostate cancer mostly affects men with a high social economic index (SDI) and longer life expectancy, who have greater access to screening and diagnosis [23]. The increasing trends of prostate cancer in Iran mainly attributed to effective diagnostic procedures, the aging of the population and a various of behavioral factors, such as smoking and Westernized diets [16,22], as well as genetic factors [24]. The healthcare system should focus on preventing the increase in the metastatic stages of the disease through early detection and timely treatment of malignancy [25].

This study revealed that stomach and prostate cancers were the leading causes of cancer mortality and DALY in most of the provinces of Iran in 2019. According to GLOBOCAN 2020 estimates, stomach cancer accounted for 7.7% of all cancer deaths and was the fourth leading cause of cancer mortality [26].

Based on the findings from the GBD, 1990–2019, the global incidence and mortality rates of gastric cancer the decreased in both sexes in the most countries of the world [16]. Also, according to GBD 2017 results, the percentage change in incidence rates of stomach cancer from 1990 to 2017 was −47·1 (95% UI: −49·0 to −45·3). In the MENA region, this value was −42·2 (95% UI: −46·9 to −36·5) (27). From 1990 to 2017, stomach cancer showed a decreasing trend in both incidence and cause of cancer related deaths worldwide. In this report indicated that 38·2% (95% UI: 21·1–57·8) of the age-standardized DALYs were attributable to a high-sodium diet, and 24·5% (95% UI: 20·0–28·9) of the age-standardized DALYs were attributable to smoking in males [27].

Lee and colleagues ‘review showed that mass eradication of H pylori infection was associated with a reduction in the incidence of stomach cancer. H pylori infection is the main known risk factor for stomach cancer [28]. To date, Helicobacter pylori infection, dietary factors, tobacco, obesity, and radiation have been identified as risk factors for stomach cancer. Therefore, reducing exposure to these risk factors, screening, and early detection, are the most important ways of preventing stomach cancer [26].

Stomach cancer is a critical and multifactorial disease in Iran. Moradian et al. reported that the incidence of gastric cancer in Iran in 2014 (13.0 × 10^5^) was higher than the worldwide rate (12.1 × 10^5^) [29]. Several factors may contribute to this trend, including behavioral factors such as, smoking, alcohol consumption, drug use, environment-genetic interaction, and lifestyle [29–30]. The incident of tracheal, bronchus, lung cancer, along with mortality rate and DALYs has been increasing worldwide. Asia has the highest of TBL cancer burden, followed by high-income North America [31]. In this study, among women, stomach cancer had the highest mortality rate and DALY in 2019, followed by tracheal, bronchus, lung, and colon, and rectum cancers. An analysis of a GBD study in 2019 in Iran showed that the incidence, mortality, and DALY rates for tracheal, bronchus, lung cancer had increased over the past 30 years [32].

Between 1990 and 2019, the tracheal, bronchus, lung cancer age-standardized death rate increased from 11.8 (95% UI: 9.7–14.4) to 12.9 (95% UI: 11.9–13.9) per 100,000 in Iran, which was higher among women [32]. Iran had an upward pattern in death and DALY rates of tracheal, bronchus, lung cancer in women, this trend could be attributed to the increasing prevalence of smoking among women [32]. In 2019, the age-standardized prevalence of smoking among individuals aged 15 and older was reported at 3.5 (95% UI: 2.8 to 4.3) for females and 37.6 (95% UI: 35.4 to 39.6) for males in Iran [33].

This increasing trend among women can be attributed to use the water-pipe (WP) smoking [34]. Air pollution is another factor contributing to the rise in deaths from tracheal, bronchus, lung cancer in Iran. Several studies indicated that PM2.5 and SO2 concentrations in Iran exceed standard pollutant values [35,36]. Benzene is another major contributor to air pollution, representing a significant health concern as it is commonly added to gasoline and is known to cause cancer [37]. The study showed that mortality and the DALY ranking for pancreatic, ovarian, and breast cancer increased in 2019 compared to 1990. Globally, from 1990 to 2017, the number of deaths, incident cases, and DALYs attributed to pancreatic cancer has more than doubled [38].

In Iran, pancreatic cancer increased about 86% between 1990–2017, nearly doubling during this period. Despite this rapid rise, Iran has a lower mortality rate compared to other countries with similar socio-demographic conditions [39]. This growth is probably related to factors such as aging [40,41], population growth [41], rising obesity or high BMI [42], diabetes [43], smoking [44], metals, textile dust, organic solvents [45], and other lifestyle and environmental factors [38]. A previous review highlighted the increasing trends in mortality and incidence of pancreatic cancer in Iran. It reported that smoking, aging, and lifestyle changes are the most important risk factors for pancreatic cancer [39]. Another review showed obvious associations between pancreatic cancer and several risk factors, including smoking, obesity, diabetes mellitus, alcohol, gender consumption, age, family history of pancreatic cancer, chronic pancreatitis, and genetic factors in the Iranian population [46]. Among all types of cancers, pancreatic cancer is highly aggressive and malignant, with a low resection rate and a very poor prognosis [47]. Besides, it lacks certain tumor markers for early diagnosis of the initial symptoms, making it difficult to diagnose early using the current imaging technologies for large-scale screening [48]. As a result, this cancer is often diagnosed late, leading to a low survival rate [39].

In this study, we observed trends in ovarian cancer that align with finding from previous studies in other regions of the world. From 1990 to 2019, the global burden of ovarian cancer increased by 107.8% (95% UI: 76.1 to 135.7%) of new cases, 103.8% (95% UI: 75.7 to 126.4%) deaths, and 96.1% (95% UI: 65.0 to 120.5%) of DALY ovarian cancer in the world [49]. Also, the study of GBD 2017 showed the same trends in the mortality burden of ovarian cancer [17]. Mortality rates based on the national registry data in Iran indicate notable increasing trends in ovarian cancer mortality and incidence in 2003–2009 [18]. Studies suggested that the high mortality and low survival rates for this cancer are primarily due to the lack of screening [50] and population aging [17]. Ovarian cancer can be attributed to multiple risk factors, for instance, a history of smoking, null parity, high fasting plasma glucose, obesity, and occupational asbestos exposure [49,51].

Regarding breast cancer trends, previous research using data from the GBD 2019 project reported that the burden of breast cancer has been increasing globally from 1990 to 2019. The age-standardized rates for incidence, death, and DALY were 24.2, 8.6, and 247.6 per 100,000, respectively, in 2019 [52]. Globally, both breast cancer deaths and DALYs nearly doubled from during this period, although the ASMR and ASDR decreased slightly, with the EAPC of −0.5 (95% UI: −0.5, −0.4) and −0.4 (95% UI: −0.5, −0.4), respectively [53]. Furthermore, the global number of female breast cancer cases increased by 128.3% between 1990 and 2019. In addition to, the fastest growth in breast cancer incidence in the MENA region [53]. Another study reported a significant increase in the incidence rate of female breast cancer in MENA over the past three decades [54]. Two similar studies have estimated the burden of breast cancer based on the GBD study 2017 [55,56].

Sharma’s study on the burden of breast cancer in Iran reported an incidence of breast cancer was 14743 (95% UI: 13248–16469), the death was 4704 (95% UI: 4306–5192), and DALY of 161486 (95% UI: 147227–177500) and the ASIR was 34 (95% UI: 30.7–37.9) in 2019. From 1990 to 2019, the breast cancer incidence and death rate rose in Iran by 28% and 81%, respectively [57]. With aging, escalating dietary and behavioral risk factors, and lower survival rates due to late-disease presentation in low- and medium-income countries, breast cancer has become a significant public health threat [57].

Although, this study showed that cancers of cervical, malignant melanoma of the skin, leukemia, and brain and central nervous system showed decreased mortality and DALY in the past three decades. Across all countries, decreasing trends were observed in the incidence, death, and DALYs of cervical cancer from 1990 to 2019 [58]. Hamdi conducted a systematic review and found that the lowest cervical cancer ASIR was observed in the MENA region, which may be due to cultural factors and conservative sexual behaviors [59]. Countries in the MENA region are conservative regarding sexual behaviors compared with the West because they have more traditional religious and social norms [60]. A meta-analysis conducted from 1990 to 2016 in Iran reported that the ASIR and ASMR of cervix cancer in Iran were 2.1 per 100,000, (95% UI: 1.9–2.4) and 0.9 per 100,000 (95% UI: 0.8–1.0), respectively. The incidence of cervix cancer in Iran is lower than that observed globally and in other countries [61]. Furthermore, Over 80% of cervical cancer deaths can be prevented through prevention strategies and screening [62]. The results of this study showed a declining trend in malignant melanoma of the skin during the last three decades. However, Yang et al. conducted a trend analysis between 1985 and 2015 using the WHO Mortality Database across 31 countries and identified an overall increase in mortality from malignant melanoma in most countries for males and more variable trends in mortality for females [63], this decreasing trend of malignant melanoma of the skin in Iran is probably complex and mostly attributed to the awareness of sun-safe behaviors and potential adverse health effects of UVR, the availability of treatments, and early diagnosis.

We found considerable inequalities between provinces with the highest and lowest rates of mortality and DALYs per100, 000 rates of all cancers. The geographical distribution of the mortality and DALYs per100, 000 rate trends of cancers was more pronounced in the northwestern and northeastern provinces of the country than in other provinces. A study in Iran reported a higher slope of concentration of PM2.5 in western provinces in Iran from 1990 to 2016 [35]. Policymakers should remain aware that the number of DALYs reflects the burden of cancers that the Iranian health system must manage.

### Strengths and limitations

A strength of our study is that we presented an updated burden of all cancers at the national level in Iran. However, this study has several notable limitations. First, the reliability of the GBD estimation data source is heavily dependent on the quality of the data used. A major limitation of the GBD study is the availability of raw data; when such data is not accessible, results depend on predictive modeling [64]. Second, our study suffers from a lack of data on risk factors related to all cancers. Future investigations in Iran should examine exposure to behavioral, occupational, and environmental carcinogens associated with various cancer types. Third, the study did not report cancer burden based on tumor site or molecular and pathological subtypes. For instance, the data did not differentiate between cancers of the trachea, bronchus, and lung, nor did it estimate the site-specific burden of colon cancer (e.g., ascending, transverse, and descending colon). Finally, this study only evaluated the cancer burden up to 2019, and the impact of the COVID-19 pandemic and long COVID on cancer burden remains unassessed. Future research should address this gap and analyze the effectiveness of cancer screening programs during pandemic times.

## Conclusion

Overall, the mortality rate and DALY per 100,000 cancers for both genders in Iran have declined from 1990 to 2019, yet there is a rising trend in various cancer types, including pancreatic, ovarian, and breast cancer. Mortality and DALYs associated with cancer increased with age, and these indicators were significantly higher in men compared to women across all age groups. Consequently, the mortality rate and DALY per 100,000 cancers were elevated in northwest and northeast Iran. Notably, stomach cancer was the leading cause of cancer-related deaths in 23 provinces of Iran in 2019. The most substantial percentage change in DALYs per 100,000 between 1990 and 2019 was observed for malignant melanoma of the skin, gastric cancer, and cervical cancer. To alleviate the national cancer burden in the future, it is crucial to implement effective and locally adapted cancer prevention and control strategies.

## Supporting information

S1 Data Changes in mortality and DALYs rates by cancer type and their ranking in each province of Iran in 1990 and 2019 by sex. (RAR)
